# Effect of low-level CO_2_ on innate inflammatory protein response to organic dust from swine confinement barns

**DOI:** 10.1186/s12995-017-0155-8

**Published:** 2017-03-24

**Authors:** David Schneberger, Jane M. DeVasure, Kristina L. Bailey, Debra J. Romberger, Todd A. Wyatt

**Affiliations:** 1 Research Service, Veterans Administration Nebraska Western Iowa Health Care System, Omaha, NE 68105 USA; 2 Pulmonary, Critical Care, Sleep & Allergy Division, Department of Internal Medicine, University of Nebraska Medical Center, 985910 The Nebraska Medical Center, Omaha, NE 68198-5910 USA; 3 Department of Environmental, Agricultural and Occupational Health, University of Nebraska Medical Center, 985910 The Nebraska Medical Center, Omaha, NE 68198-5910 USA

**Keywords:** Carbon dioxide, Hypercapnia, Barn dust

## Abstract

**Background:**

Organic hog barn dust (HDE) exposure induces lung inflammation and long-term decreases in lung function in agricultural workers. While concentrations of common gasses in confined animal facilities are well characterized, few studies have been done addressing if exposure to elevated barn gasses impacts the lung immune response to organic dusts. Given the well documented effects of hypercapnia at much higher levels we hypothesized that CO_2_ at 8 h exposure limit levels (5000 ppm) could alter innate immune responses to HDE.

**Methods:**

Using a mouse model, C57BL/6 mice were nasally instilled with defined barn dust extracts and then housed in an exposure box maintained at one of several CO_2_ levels for six hours. Bronchiolar lavage (BAL) was tested for several cytokines while lung tissue was saved for mRNA purification and immunohistochemistry.

**Results:**

Exposure to elevated CO_2_ significantly increased the expression of pro-inflammatory markers, IL-6 and KC, in BAL fluid as compared to dust exposure alone. Expression of other pro-inflammatory markers, such as ICAM-1 and matrix metalloproteinase-9 (MMP-9), were also tested and showed similar increased expression upon HDE + CO_2_ exposure. A chemokine array analysis of BAL fluid revealed that MIP-1γ (CCL9) shows a similar increased response to HDE + CO_2_. Further testing showed CCL9 was significantly elevated by barn dust and further enhanced by CO_2_ co-exposure in a dose-dependent manner that was noticeable at the protein and mRNA levels. In all cases, except for ICAM-1, increases in tested markers in the presence of elevated CO_2_ were only significant in the presence of HDE as well.

**Conclusions:**

We show that even at mandated safe exposure limits, CO_2_ is capable of enhancing multiple markers of inflammation in response to HDE.

## Background

Concentrated animal feeding operations (CAFOs) are a common feature of agriculture in developed countries. Exposure to the organic dusts within these facilities has been shown to cause a number of short and long-term problems for exposed workers. These problems include, but are not limited to, increased risk for asthma and COPD [1–3], chronic bronchitis [4], and a general decease in lung function over time exposed [5].

The immune response to organic barn dusts is mediated primarily by the innate immune system. Previous work identifies endotoxin and peptidoglycans as key components of the dust that trigger innate immune responses in humans, cell cultures, and mouse models [6–9]. These bacterial components act through the TLR4 and TLR2 receptors, respectively [10, 11], and it has been shown that one or both of these receptors may be critical to the immune response generated to these dusts [10–12], though it is doubtful that these components are the only causes of this inflammation.

Response to hog barn dust extracts (HDE) typically involves expression of pro-inflammatory cytokines and chemokines such as TNF-α [8, 13], IL-6 [13–15], and IL-8 (KC in mice) [13–16]. This increased cytokine expression, particularly through IL-8, results in increased infiltration of cells, particularly neutrophils, into the lung [9, 16]. Other changes to the lung will occur such as increased edema, and prolonged exposure can lead to lymphoid aggregate formation [9]. A host of other chemokines will also likely be induced in this response, but IL-6 and KC are the most often characterized markers of this inflammation.

HDE exposure does not occur in isolation of gasses in the barn air, and three gasses, in particular, are commonly recognized as being elevated in most CAFO operations: ammonia (NH_3_), hydrogen sulfide (H_2_S), and carbon dioxide (CO_2_). Ammonia has potential negative effects on lung function and health of workers and animals in the CAFO environment [17, 18] though little is known. H_2_S is recognized as a serious potential health hazard in CAFO operations, usually involving waste management and removal. H_2_S in these workplace exposures is both an irritant and an asphyxiant, and if levels are high and sustained, fatal.

CO_2_, on the other hand, may be elevated as high as 5000 ppm in some facilities [19], but perceived effects are often mild and do not include irritation [20]. As such, it is often not considered a potential problem in the barn environment and has thus received little to no study. Studies with cell cultures and animal models have yielded mixed results as to the pro- or anti-inflammatory nature of hypercapnia [21–23]. In almost every case, the levels used in these studies far exceed CO_2_ levels that may be encountered in a workplace environment. We hypothesized that the combination of CO_2_ gas exposure enhances the inflammatory response to HDE exposure.

Given that elevation of other gasses in this environment showed potential to enhance lung inflammatory symptoms [18], that CO_2_ could alter response to dust extract exposures in bronchial epithelial cells [24] and that levels as low as 1000 ppm were capable of inducing changes in cognitive function [25], we hypothesized that the elevation of CO_2_ gas exposure may enhance the inflammatory response to HDE exposure. We therefore looked to see if there was any effect of CO_2_ on HDE exposure at the Occupational Safety and Health Administration (OSHA) 8 h workplace permissible exposure limit of 5000 ppm. Using a mouse model system of HDE exposure, we show that even at this workplace allowable limit there were significant changes to IL-6, KC, and CCL9. Further examination of CCL9 showed that the chemokine was induced by HDE, and increased in a dose-specific manner to co-exposure with elevated CO_2_ at the mRNA and protein levels. As CCL9 is suggested to potentially have an effect on MMP-9, we tested and found a similar pattern in relation to MMP-9 mRNA expression. Another potential marker of enhanced inflammation in the lung, ICAM-1, may be increased or decreased during hypercapnia [26, 27]. Thus, we tested for ICAM-1 expression in mice and found a similar increase in response to HDE plus CO_2_ in lung and trachea.

We therefore show that 8 h CO_2_ permissible exposure levels can enhance the stimulated expression of several cytokines, chemokines, and pro-inflammatory markers in the lung in response to organic barn dusts.

## Methods

### Hog barn dust extracts

HDE extracts were prepared from settled dust samples combined from two separate swine confinement facilities and have been characterized as to content of protein, endotoxin, and muramic acid [28]. The bacterial composition has also been characterized [29]. Dust extracts were prepared as previously described [15]. Dust (1 g) was mixed with 10 ml HBSS without calcium and incubated for 1 h at room temperature before centrifugation for 10 min, with media being decanted and sterile-filtered for use, for a final concentration of approximately 0.105 g/ml dust. No stability problems are noted in such extracts for at least a year or more after storage. Extracts were used at a concentration of 12.5% v/v or about 0.005 g/ml dust and prepared no earlier than 2 weeks before use.

### Animals

All procedures were approved by the Institutional Animal Care and Use Committee of the University of Nebraska Medical Center (protocol number 04-059-08). Female, 6–8-week-old C57BL/6 mice (Charles River, Wilmington, MA) were acclimated for one week after arrival. The animals were group-housed, and their diet consisted of commercial rodent chow and water *ad libitum*. Animals were assigned randomly to each treatment group: saline, saline + hypercapnia, HDE instillation (12.5%), or HDE instillation + hypercapnia. All mice (8 per group) were instilled nasally one time [11] with 40 μl of treatment. Mice were treated for 6 h in an exposure chamber, with controlled fresh air ventilation and a fan to circulate air within the box. Mice exposed to elevated CO_2_ were given CO_2_ in the same box. Levels of CO_2_ were assayed using a CO_2_ monitor (Extech CO210, Extech, Nashua, NH) and the chamber was manually ventilated to maintain levels for normal CO_2_ (400 ppm), and hypercapnia (5000 or 7500 ppm). All treatment groups were split over two separate occasions, separated by no more than two weeks (4 animals/group/occasion; *n* = 8).

Sacrifice of animals was done within 1 min of withdrawal from exposure chamber, and staged so that BAL and lung excision did not exceed 30 min after completion of treatment.

### Serum collection

Blood was taken from animals at time of sacrifice by cardiac puncture in serum collection tubes (Microtainer, Becton Dickson, Franklin Lakes, NJ) using an 18-gauge needle (Becton Dickson). Samples were centrifuged 10 min at 7000 × g and serum stored at -80 °C until used for ELISA. Blood was not taken from the 7500 ppm CO_2_ exposed group by mistake.

### Bronchoalveolar lavage (BAL) collection

Lungs were lavaged as detailed previously [9]. Briefly, lungs were washed three times with 1 ml sterile saline each time. BAL fluid was centrifuged 1750 × g for 10 min and supernatant samples stored at -80 °C until used. Cells were resuspended in 1 ml PBS, counted, and 1.5 × 10^3^ cells adhered to glass slides via cytospin. Cells were stained using a Diff-Quik kit (Siemens Healthcare Diagnostics, Newark, DE) and cover slips mounted. A differential count of at least 200 cells was made based on morphometric criteria and expressed as absolute cell numbers (mean +/- SEM).

### Lung collection

After BAL collection, lungs were excised from animals. The left lung was tied off at the primary bronchus and removed, flash frozen in liquid nitrogen and stored at -80 °C for mRNA collection. A cannula was inserted in the trachea of the remaining lung and cinched with a suture. Lungs were hung in a bath of 10% formalin fixative as 0.8 ml of this fixative was allowed to enter the lungs via the cannula for 24 h under a pressure of 15 cm H_2_O. The fixed lung was then embedded in paraffin for later immunohistochemical staining.

### Tracheal cell collection

A separate exposure was done with 10 mice/group at normal CO_2_ (400 ppm), and hypercapnic (7500 ppm) CO_2_ levels. The trachea was saved from each animal, opened, and the internal surface scraped gently with a cell scraper into cell lysis buffer (Qiagen, Chatsworth CA) and processed for RNA as per whole lung tissue (described below). Cell lysate samples were pooled from 2 mice to yield enough mRNA for testing.

### ELISAs

Cytokine and chemokine quantitation of BAL fluid was done by enzyme linked immunoabsorbant assay kits to IL-6, KC, and CCL9 (R&D Systems, Minneapolis, MN) according to manufacturer’s instructions. Broad spectrum testing of BAL fluid for chemokine expression was accomplished using a dot blot array kit (ARY020, R&D Systems, Minneapolis, MN).

### Wet/Dry ratio

Four additional mice per group were exposed as described previously and lungs removed after treatment. Lungs were weighed at time of removal and then dried overnight in a drying oven at 60 °C and visually checked for dryness at the end of this time before being re-weighed. Ratio was calculated of wet to dry weight.

### RNA purification and RT-PCR analysis

RNA was purified from lung tissue samples using a Qiagen spin miniprep kit according to manufacturer’s instructions, including additional DNAse digestion (Qiagen, Chatsworth CA). Initial homogenization of tissue was done in 350 μl of lysis buffer from the miniprep kit with 2.0 mm stainless steel beads (Next Advance, Averill Park, NY) and placed in a Bullet Blender Storm 24 magnetic bead beater (Next Advance, Averill Park, NY) for 3 m at 4 °C. RNA was quantified by NanoDrop ND-1000 (Thermo Scientific, Wilmington, DE).

cDNA synthesis was performed using the Taqman reverse transcription kit (Applied Biosystems, Branchburg, NJ) with 200 ng of template mRNA. cDNA synthesis (RT-PCR) reactions contained the following reagents: 1X TaqMan RT buffer, 5.5 nM MgCl_2_, 500 μM of each dNTP, 2.5 μM random hexamers, 0.4 U/μl RNase inhibitor, and 1.25 U/μl MultiScribe reverse transcriptase as per kit instructions (Applied Biosystems, Branchburg, NJ). Samples were incubated at 25 °C for 10 min, then 48 °C for 30 min, and 95 °C from 5 min in a thermocycler (MJ Mini; Bio-Rad, Hercules, CA).

RT-PCR was performed using probes to CCL9 (Life Technologies, Mm00441260), MMP-9 (Mm00600163), and ICAM-1 (Mm00516023). Ribosomal RNA was used as an endogenous control. PCR was conducted using an ABI PRISM 7500 Sequence Detection System (Applied Biosystems). Reactions were carried out for 2 min at 50 °C, 10 min at 95 °C, followed by 40 cycles at 95 °C for 15 s and 60 °C for 1 min each. All reactions were carried out in duplicate. For relative comparison of targets to the ribosomal RNA endogenous control, we analyzed cycle threshold (CT) value of real-time PCR data with the ∆∆Ct method.

### ICAM-1 tissue staining

ICAM-1 staining was localized in lung tissue as previously reported [9]. Briefly, tissue was blocked overnight at 4 °C, with primary antibody added at 1:75 (rat anti-mouse ICAM-1; Rockland Immunochemicals, Gilbertsville, PA) and incubated for 1 h at room temperature, followed by secondary antibody (rat anti-CD54, 1:300; Biolegend, San Diego, CA) for 2 h.

### Statistical analysis

All data was analyzed using GraphPad Prism (GraphPad Software, San Diego, CA). Error bars represent the mean +/- SEM. Statistical significance was determined using ANOVA with follow-up Bonferroni test, with *p* ≤ 0.05 confidence interval being considered significant.

## Results

### BAL cell numbers are unaffected by hypercapnia

**Fig. 1:**
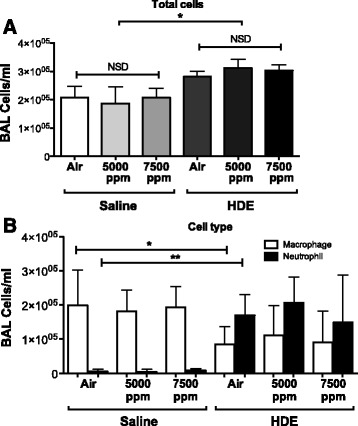
Effect of HDE on BAL cell counts. Exposure to HDE caused an increase in total BAL cell numbers (**a**) and a shift from predominantly macrophages to neutrophils (**b**). Increasing CO_2_ levels (5000 and 7500 ppm) from ambient air (air) had no significant effect on BAL cell number or type in BAL samples. Bar graphs represent standard deviation with error bars shown (*N* = 8 mice/group, *N* = 4 mice/group for 7500 ppm groups). Statistical significance denoted by asterisks (*p < 0.05, **p < 0.01) as compared to respective saline treatment group

Counts of cells harvested from BAL of mouse lungs showed that nasal instillation of barn dust induced an increase in total cell numbers in the lung (Fig. 1a), and a shift in the population from predominantly macrophages to predominantly neutrophils (Fig. 1b), as has been seen in previous studies [9, 24]. Additional exposure to elevated CO_2_ conditions induced no discernable changes in either number or type of cells present in the alveolar space, both in saline as well as barn dust exposed animals, even when CO_2_ was increased to 7500 ppm.

### Cytokine expression in response to barn dust is Increased with hypercapnia

**Fig. 2:**
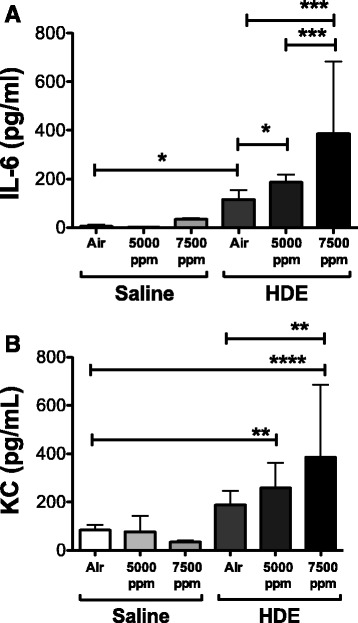
CO_2_ enhances HDE stimulated cytokine expression in BAL. BAL fluid was tested by ELISA for (**a**) IL-6 and (**b**) KC. HDE instillation induces both cytokines. Increasing CO_2_ levels (5000 and 7500 ppm) in HDE-treated mice significantly increased production of both cytokines vs ambient air (air). Bar graphs represent standard deviation with error bars shown (*N* = 5-8 mice/group). Statistical significance denoted by asterisks (*p < 0.05, **p < 0.01, ***p < 0.001, ****p < 0.0001)

BAL was tested first for common markers of inflammation, IL-6 and KC. Both IL-6 (Fig. 2a) and KC (Fig. 2b) were increased in response to barn dust as has been reported previously [5, 13–15] though only IL-6 was significantly elevated by six hours. Exposure to 5000 and 7500 ppm CO_2_ caused significant increases in both IL-6 and KC in HDE-exposed animals compared to saline. This effect was only seen with exposure to HDE, as saline-treated mice showed no similar cytokine increases in response to elevated CO_2_. No changes were noted in the serum for either (results not shown).

**Fig. 3:**
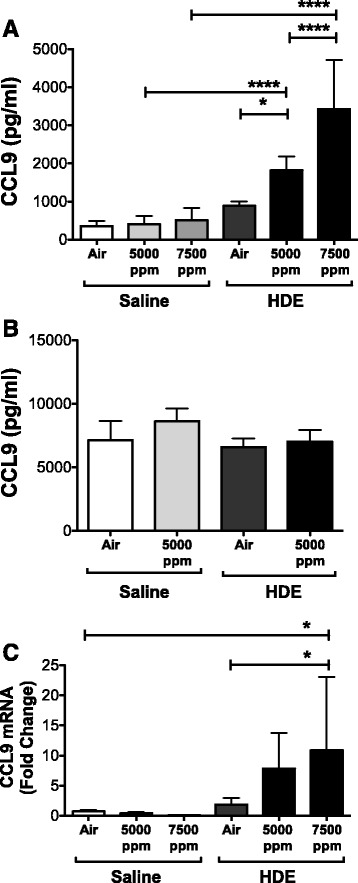
CCL9 is increased in response to HDE and CO_2_. Expression of CCL9 chemokine was examined in BAL (**a**) and serum (**b**) by ELISA. Only the lung showed significant increases in CCL9 in response to HDE. Increase of CO_2_ (5000 and 7500 ppm) induced what appeared to be a concentration-dependent increase in CCL9 production vs. ambient air (air) in the lung that was absent in serum as well. Expression in lung was confirmed by mRNA expression (**c**) which showed a similar pattern of CCL9 induction to barn dust and CO_2_. Bar graphs represent standard deviation with error bars shown (*N* = 6-8 mice/group). Statistical significance denoted by asterisks (*p < 0.05, **p < 0.001, ***p < 0.0001)

To look for other possible chemokine targets that may be altered in response to the combination of HDE with elevated CO_2_, we tested BAL samples using a chemokine dot blot array. Numerous chemokines were altered by HDE (data not shown), however one chemokine, CCL9 showed what appeared to be a noticeable change in response to CO_2_. To confirm this result, we tested BAL for CCL9 using ELISA to quantitate the results (Fig. 3a). CCL9 expression was significantly increased by exposure to HDE, and significantly increased over those levels when mice were exposed to HDE plus CO_2_. The levels of CCL9 were increased further still at 7500 ppm CO_2_ plus HDE, though none of these increases was reflected in serum samples (Fig. 3b), which were uniformly high, as has been shown in other studies [30]. Examination of mRNA showed that increase in CCL9 occurred at the mRNA level as well in lung tissue (Fig. 3c).

### CO_2_ does not induce significant lung leak

**Fig. 4:**
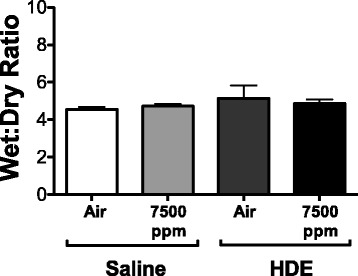
Mean wet:dry ratio of mouse lung after HDE instillation and/or CO_2_ (7500 ppm) treatment. Lungs were weighed post-necropsy, desiccated and weighed to determine wet:dry ratios to examine lung leak. No significant changes from ambient air (air) were noted for any treatments. Bar graphs represent standard deviation with error bars shown (*N* = 3 mice/group)

As increased CCL9 levels in the lung may reflect increased leak from the blood, additional mice were treated as in previous experiments, and whole lungs were collected for measure of wet-to-dry ratio. There was no significant increase in wet-to-dry ratio of the lungs of HDE treated or HDE + CO_2_ (7500 ppm) treated animals, suggesting extra fluid had not accumulated in the lungs due to leak (Fig. 4).

### ICAM-1 expression

**Fig. 5:**
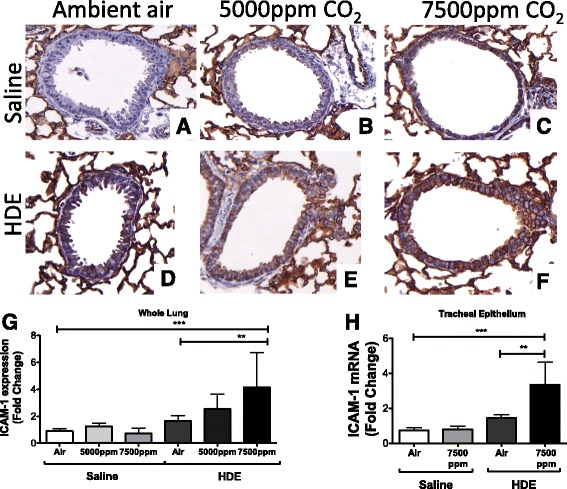
ICAM-1 Expression in bronchial epithelium is altered by HDE and CO_2_ exposure. Saline treated mice (**a-c**) showed increased ICAM-1 staining at 5000 ppm (**b**) and 7500 ppm CO_2_ (**c**). The same pattern was observed in HDE-treated mice (**d-f**) with ambient air (**d**), 5000 ppm CO_2_ (**e**), or 7500 ppm CO_2_ (**f**). ICAM-1 mRNA harvested from whole lung (**g**) and tracheal epithelium (**h**) showed no significant increases in response to CO_2_ exposure, but HDE with CO_2_ treatment induced significant increases of ICAM-1 mRNA. Bar graphs represent standard deviation with error bars shown (5G *N* = 7-8 mice/group, 5H *N* = 4 mice/group). Statistical significance denoted by asterisks (*p < 0.05, **p < 0.01, ***p < 0.001)

ICAM-1 expression is reported to be either increased [26] or decreased [27] in response to hypercapnia. To address this, we performed immunohistochemical staining of mouse lungs. We observed a visible increase in ICAM-1 expression in the bronchial epithelium exposed to saline + 5000 ppm CO_2_ alone, which was even more pronounced at 7500 ppm (Fig. 5a–c). ICAM-1 expression was also increased by HDE alone. The addition of CO_2_ exposure caused further increases (Fig. 5d–f). To quantitate these visual observations, we examined ICAM-1 mRNA expression. CCL9 mRNA expression in whole lung homogenates showed a similar pattern to what was seen with immunohistochemical staining (Fig. 5g). There was no change in ICAM-1 mRNA in saline-treated animals with increased CO_2_, but in HDE + CO_2_ treated animals, there were increases in mRNA which became significantly elevated at the 7500 ppm level over that of HDE treatment alone. This was true in both whole lung (Fig 5g) as well as tracheal epithelium (Fig. 5h) for mRNA samples.

### MMP-9 mRNA expression

**Fig. 6:**
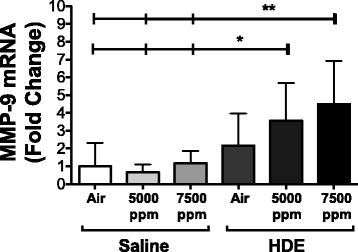
HDE plus CO_2_ induces MMP-9 mRNA expression. Whole lung mRNA was tested for MMP-9 expression. While HDE alone did not increase MMP-9 mRNA expression, the addition of 5000 and 7500 ppm CO_2_ induced significant increases. Bar graphs represent standard deviation with error bars shown (*N* = 8 mice/group). Statistical significance denoted by asterisks (*p < 0.05, **p < 0.01) as compared to all saline treatment groups

A feature of obstructive lung disease is an increase in MMP-9. We suspected that hypercapnia plus an inflammatory stimulus may have a similar effect. Testing for mRNA expression in whole lung mRNA showed that MMP-9 was not increased by hypercapnia alone, and HDE treatment alone did not induce a significant increase in mRNA (Fig. 6). Combined HDE + hypercapnia treatment, however, showed a significant elevation of MMP-9 mRNA that appeared to be CO_2_ dose-dependent (Fig. 6).

## Discussion

Workers in CAFOs are exposed to a mixture of organic dusts and gasses in barns. These gasses are produced from the animals living in the building as well as gasses released from wastes and bacterial action. While changes to ventilation may impact dust and gasses in the air, other dust remediation steps such as cleaning or sprinkling [12] may reduce dust, but not gasses. These problems may be greater in colder climates where ventilation must be balanced against heat loss and energy costs.

Of the gasses often studied in CAFOs, three are commonly increased as a result of biological processes in these facilities; NH_3_, H_2_S, and CO_2_ [31–33].

CO_2_, while known to cause headaches at higher levels has no current association with known occupational disease. CO_2_ is a commonly elevated gas in some work environments such as wastewater treatment [34], and potentially small airtight spaces with several people in them [35]. Many well-ventilated facilities will not reach the 8 h time weighted average exposure (TWA) OSHA limit (5000 ppm), but some facilities tested have been shown to reach these levels [33]. Work by other groups into the effects of permissive hypercapnia show CO_2_ alters immune responses to treatments such as endotoxin [36], however the amounts used in many of these experiments are levels not applicable to work exposures. This study addresses this deficiency by examining relatively low dose CO_2_ in whole animals using a workplace-relevant inflammatory stimuli (organic barn dust).

Initial examination of cells in the BAL showed HDE treatment increased cell number as well as the number of neutrophils, as has been shown previously [5, 13–15]. Exposure to hypercapnic conditions had no effect on either number or type of cells present other than a mild non-significant increase in total cell numbers in the lung.

When we looked at the release of inflammatory cytokines/chemokines IL-6 and KC, with or without HDE under hypercapnic conditions, hypercapnia had a clear effect. Both cytokines are associated with lung inflammation [14, 15] and in our studies, both were significantly increased in response to HDE + CO_2_ exposure. As we did not see a similar increase in saline + CO_2_ exposures, this suggests that hypercapnia may alter response to an inflammatory stimuli such as barn dust while not necessarily inducing some of these responses by itself. What the mechanism is behind such an alteration remains unknown and is the focus of our ongoing studies. The response to HDE may also be changed in other subtle ways. KC is a strong neutrophil chemotactic chemokine [37], and while it is clearly increased in response to HDE + CO_2_, we did not see any difference in neutrophil migration.

CCL9 is produced primarily by macrophage cells [38] and typically exist at high constitutive levels in the blood [30]. While little work has examined CCL9 in the lung, a silicosis model shows that challenge with silica induced a specific increase of lung CCL9 [39]. There too they noted very low BAL levels of CCL9 outside of stimulation with particulates. Another paper notes that injection of endotoxin can induce an increase of CCL9 in the heart and lung [30], a key component of barn dusts. In the current study we show that not only is CCL9 induced by HDE, but that it is significantly increased in a CO_2_ dose-dependent manner when given with HDE. Given the high constitutive expression of CCL9 in circulation, there was concern that an increase in epithelial leak may be responsible for the elevation in CCL9 in BAL samples. Wet-to-dry ratios of the lungs, however, were unaffected, showing increased lung leak was not a factor. More telling, however, mRNA from lung tissue showed an increase that generally reflected protein levels seen in the lung, suggesting that CCL9 present in BAL is locally produced in response to HDE and HDE + CO_2_ while serum levels of protein were unaffected.

CCL9 is chemotactic for Langerhans cells and CD4+ T cells in other tissues [38, 40]. The chemokine binds the CCR1 receptor [30], but is less chemotactic than other ligands for this receptor. The mechanisms by which CCL9 functions are still mostly unresolved, though local production in response to challenge suggests some potential function in the response to inhaled challenges to the immune system. It was discovered that proteolytic cleavage of CCL9 greatly enhances its chemotactic potential [41], suggesting that CCL9 may act as an early response chemokine, responding to local proteolysis that enhances the chemotactic signal. This is of particular interest to our work in that our group has recently demonstrated that HDE is capable of inducing proteolytic cleavage/activation of PAR receptors [42] by HDE. It is therefore possible that CCL9 may be similarly activated in the alveolar space in response to barn dust. We are currently examining this possibility.

Another important marker of increased inflammation is ICAM-1 expression. This cell receptor is vital for migration of neutrophils to the lung [43], and given the lack of significant neutrophil increase, we wondered if CO_2_ had any effect on this receptor. Somewhat surprisingly, we did see clear increases in tissue staining for the receptor in the bronchial epithelium with saline + CO_2_ alone. Because HDE predictably induced ICAM-1 so significantly in these bronchial cells [44], it was not clear if addition of CO_2_ enhanced this effect or not. An examination of lung mRNA showed that mRNA was indeed increased with HDE + CO_2_, but not so with saline + CO_2_. As whole lung RNA samples may not reflect tracheal mRNA we isolated tracheal epithelial cell mRNA from animals as well, showing a similar pattern of expression to whole lung samples. This may suggest that a process in ICAM-1 protein production may be responsible for the increases we see with CO_2_ treatment alone. This may also help to explain the modest increase in ICAM-1 mRNA with HDE treatment alone, despite its clear increase in response to HDE [44].

Finally, we decided to look at MMP-9 due to mention of an unpublished observation that CCL9 increased MMP-9 in the lung [39] and its roles in neutrophil migration and tissue remodeling in the lung [45, 46]. Similar to the other chemokines we examined, there was a clear increase in MMP-9 mRNA in lung tissue as CO_2_ was increased. Similar to other factors examined such as CCL9, this increase was only apparent when HDE was present.

## Conclusions

These results raise a number of questions with relation to workplace ventilation. Does ventilation need to be considered with relation to common illnesses and contaminants of workplaces and how they may interact? While elevated CO_2_ exposure alone in most cases appeared insufficient to induce changes, the lung appears to alter several responses when elevated CO_2_ is present in addition to an innate immune stimulus. In this respect, CO_2_ might function as a tuning mechanism of innate immunity, or perhaps an indicator of dysfunction, requiring a different or elevated response. Another question remaining regards possible acidosis in the exposed animals, and if this is a factor in altered immune responses. While blood gas sampling was not done, the anesthesia protocol at the end of the experiment had animals out of the chamber long enough to likely skew such readings. This remains a technical issue to address in future studies. What our work clearly does show is that the effects of gas exposures at levels seen in work environments, particularly in CAFOs, can depend on responses to other elements present, in particular organic dust.

While this work is in mice and uses an established laboratory-optimized dust injury model, we do note that dust extracts may not mimic a true dust exposure as encountered in the barn. Work will also have to be done with regards to which signaling pathways are affected by these exposures. We do still feel this work shows the importance of further work in mixed environmental exposures and for testing of workers to dusts and gas exposures in the work environment.

## Abbreviations

BAL: Bronchoalveolar lavage
CAFO: Concentrated animal feed operation
CCL9: CC chemokine ligand 9
CO_2_: Carbon dioxide
COPD: Chronic obstructive pulmonary disease
H_2_S: Hydrogen sulfide
KC: Keratinocyte chemoattractant
MMP-9: Matrix metalloproteinase-9
NH_3_: Ammonia
TLR: Toll-like receptor
